# Safety voice for ergonomics (SAVE) project: protocol for a workplace cluster-randomized controlled trial to reduce musculoskeletal disorders in masonry apprentices

**DOI:** 10.1186/s12889-016-2989-x

**Published:** 2016-04-27

**Authors:** Laurel D. Kincl, Dan Anton, Jennifer A. Hess, Douglas L. Weeks

**Affiliations:** College of Public Health and Human Sciences, Oregon State University, Corvallis, Oregon USA; Department of Physical Therapy, Eastern Washington University, Spokane, Washington USA; Labor Education and Research Center, University of Oregon, Eugene, Oregon USA; Department of Rehabilitation Research, St. Luke’s Rehabilitation Institute, Spokane, Washington USA

**Keywords:** Ergonomics, Safety voice, Construction industry, Masons, Apprenticeship training, Injury prevention

## Abstract

**Background:**

Masons have the highest rate of overexertion injuries among all construction trades and rank second for occupational back injuries in the United States. Identified ergonomic solutions are the primary method of reducing exposure to risk factors associated with musculoskeletal disorders. However, many construction workers lack knowledge about these solutions, as well as basic ergonomic principles. Construction apprentices, as they embark on their careers, are greatly in need of ergonomics training to minimize the cumulative exposure that leads to musculoskeletal disorders. Apprentices receive safety training; however, ergonomics training is often limited or non-existent. In addition, apprenticeship programs often lack “soft skills” training on how to appropriately respond to work environments and practices that are unsafe. The SAVE program – **SA**fety **V**oice for **E**rgonomics – strives to integrate evidence-based health and safety training strategies into masonry apprenticeship skills training to teach ergonomics, problem solving, and speaking up to communicate solutions that reduce musculoskeletal injury risk. The central hypothesis is that the combination of ergonomics training and safety voice promotion will be more effective than no training or either ergonomics training alone or safety voice training alone.

**Methods/design:**

Following the development and pilot testing of the SAVE intervention, SAVE will be evaluated in a cluster-randomized controlled trial at 12 masonry training centers across the U.S. Clusters of apprentices within centers will be assigned at random to one of four intervention groups (*n* = 24 per group): (1) ergonomics training only, (2) safety voice training only, (3) combined ergonomics and safety voice training, or (4) control group with no additional training intervention. Outcomes assessed at baseline, at the conclusion of training, and then at six and 12 months post training will include: musculoskeletal symptoms, general health perceptions, knowledge of ergonomic and safety voice principles, and perception and attitudes about ergonomic and safety voice issues.

**Discussion:**

Masons continue to have a high rate of musculoskeletal disorders. The trade has an expected increase of 40 % in the number of workers by 2020. Therefore, a vetted intervention for apprentices entering the trade, such as SAVE, could reduce the burden of musculoskeletal disorders currently plaguing the trade.

**Trial registration:**

ClinicalTrials.gov Identifier: NCT02676635, 2 February 2016

## Background

Work-related musculoskeletal disorders (MSDs) continue to be widespread in the construction industry, and brick and block masons (masons) are among the most affected of all construction workers [1, 2]. According to the 2013 Construction Chartbook [3], MSDs have dropped in number in construction. Yet, they are still 16 % higher than all other industries combined. Among all construction trades, masonry has the highest reported rate of overexertion injuries, with 66.5 injuries per 10,000 full time equivalents (FTE), the highest rate of repetitive motions, and is ranked second among the construction trades for back injuries, with a rate of 45.3 injuries per 10,000 FTE [1]. The majority of overexertion injuries among masons involve the low back, resulting in the highest associated costs of medical care [2, 4–6]. Shoulder disorders are the second most common MSD among masons, with approximately 50 % of masons complaining of shoulder symptoms [4, 5, 7].

These high injury rates are associated with the demanding physical nature of masonry work. [3, 8] Ergonomic training is one solution that can assist masons in reducing exposure to risk factors associated with MSDs [8, 9]. However, many construction workers lack knowledge about ergonomic solutions, as well as basic risk factors associated with MSDs. As trade workers embarking on their career, apprentices need ergonomics training in order to recognize and minimize the cumulative exposure that leads to MSDs, which may shorten their career and increase the likelihood of permanent disability. In response, the National Institute for Occupational Safety and Health (NIOSH) recently implemented the “Safe – Skilled – Ready Workforce Initiative” [10] focused on teaching workers to recognize and prevent work-related injuries and illnesses. In conjunction with this initiative, construction apprentices take safety training, such as the Occupational Safety and Health Administration (OSHA) 10-h course. However, ergonomics training is limited to non-existent in these educational efforts.

Training in ergonomic principles is not the only content missing from apprenticeship training. Apprentices often lack applied or soft skill training on how to appropriately respond to work environments and practices that are unsafe. Due to the hierarchical nature of apprenticeship training, young workers may face peer pressure from journey level workers to conform to the ‘way things have always been done.’ On the other hand, apprentices have the potential to embrace new concepts prior to being hardened in traditional ways of working. To address this conflict, apprentices may benefit from training on soft skills such as self-direction, self-control, accountability, responsibility, communication strategies, and leadership [11] that can help them to develop a “safety voice” about safety in general, and ergonomics specifically.

The **SA**fety **V**oice for **E**rgonomics (SAVE) program proposes to integrate progressive health and safety training strategies into the masonry apprenticeship program to teach both hard skills of ergonomics and soft skills of problem solving and speaking up to reduce musculoskeletal injury risk. SAVE will incorporate *blended learning* principles. Blended learning combines traditional, face-to-face teaching methods with e-learning methods [12–14]. For the purpose of this research program, “e-learning” is defined as training delivered by computers, tablets, or smartphones using “e-tools” such as online, interactive training units and short message service (SMS, text messaging).

Most apprentices are currently taught using traditional educational methods. In contrast, many secondary and higher education institutions are using e-learning (e.g., online courses) or blended learning methods to a greater extent, and e-learning has been demonstrated to be equally or more effective than face-to-face methods [15–19]. There is evidence that blending traditional and e-learning is effective [18], especially when presenting declarative knowledge such as occupational safety content [20]. Because of this, and since approximately 95 % of those the age of a typical apprentice use the internet and have computer access [21, 22], SAVE will use a blended approach with e-learning content followed by applied “shop” activities. This approach will make an innovative contribution to current International Masonry Institute (IMI) training and education apprenticeship curricula and could easily be adapted for other trades. IMI provides training to union masonry apprentices at the national training center as well as at over 39 International Union of Bricklayers & Allied Craftworker training centers across the US, thus, the impact of SAVE could be significant.

Other factors will be incorporated into the SAVE training materials in order to optimize training effectiveness, such as learner engagement, frequency of delivery, and a participatory process. Highly engaging and interactive training is more effective than low engagement training [17, 23, 24]. Further, multiple training sessions are most effective for knowledge retention [25] although training effects have been shown in a single session [24, 26, 27]. SAVE will employ a strategy of multiple, brief, online training units.

It is well established that knowledge decays if not used [28]. Therefore, refresher training (e.g., secondary or booster training) is essential for retention. Various types of refresher training have been used in occupational health [29, 30], but the optimal frequency or duration for refresher sessions following primary training has not been established [31]. Regardless, social media, such as text messaging or email, is an efficient method to deliver refresher training. Text messages or email have been used by public health practitioners for various purposes including smoking cessation, communicable disease awareness, and occupational safety reminders [32, 33]. Additionally, 88 % of adults in the United States use a cell phone regardless of race or ethnicity, and 91 % of adults aged 18–29 years of age use email regularly [22]. Therefore, text messaging and emails will be integrated into the SAVE training as a means of delivering ongoing refresher training.

The long-term outcome of the SAVE training is the reduction of MSDs among masons. The intermediate outcomes are improvement of 1) apprentice knowledge about ergonomics to increase the adoption of trade-specific solutions, and 2) apprentice ability to speak up about ergonomics and safety issues, that is, using a safety voice. Following development and integration of the SAVE training into existing apprenticeship training, we will measure the effectiveness of the SAVE training using a cluster-randomized control trial to test the components and the full effect of the program among masonry apprentices. Our central hypothesis is that the SAVE training will be more effective than ergonomic training alone, safety voice training alone, or no additional training. Our rationale is that this novel combination of training methods and topics will expand previous efforts to promote ergonomics and reduce MSDs by encouraging masonry apprentices to develop their ‘safety voice’ and to promote the use of ergonomic tools and work practices.

## Methods/design

### Trial design

The SAVE project is a four arm, randomized-controlled study. The units of analysis will be individual apprentices. However, the units from which the apprentices will be recruited are apprenticeship training centers, with clusters of apprentices within centers assigned at random to one study arm, resulting in a cluster randomized-controlled trial design. Training centers will be randomly assigned to one of four groups: 1) ergonomic training alone, 2) safety voice training alone, 3) combined ergonomic and safety voice training, or 4) control group with no additional training intervention (Table 1).

**Table 1:** Study design for Safety Voice for Ergonomics (SAVE) training intervention

		Safety voice training	
		Yes	No
Ergonomics training	Yes	All training	Ergonomic
	No	Safety voice	No training

An additional factor in the design will be repeated measurement of outcomes per apprentice on four occasions: 1) At baseline, 2) Post primary training for each group, 3) 6 months following primary training, and 4) 12 months following primary training. The resulting overall design will be a 2 x 2 x 4, Ergonomic Training by Safety Voice Training by Time of Measurement factorial design with repeated measures on the last factor and apprentices nested within training centers.

### Trial status

The SAVE project has been approved by the Institutional Review Board (IRB) of Eastern Washington University, the IRB of record for this project. The development of the SAVE training is underway and the pilot testing of training materials is scheduled to occur in the first quarter of 2016. Initiation of the randomized trial is expected to begin in the third quarter of 2016.

### Partnerships

This project has the support of a collaborative arrangement with the Masonry Research to Practice Partnership (Masonry r2p Partnership), whose members include the International Council of Employers of Bricklayers and Allied Craftworkers (ICE), the International Union of Bricklayers and Allied Craftworkers (BAC), and the IMI. These groups represent roughly 160 instructors, 10,000 contractors, and 100,000 craft workers across the US, including workers from masonry brick, block, tile, marble, terrazzo, stone, plaster, cement, masonry restoration/tuckpointing, and refractory work. With the Masonry r2p Partnership framework, this program will be disseminated broadly and put into practice.

### Study population

Approximately 96 currently employed masonry apprentices in their first two years of apprenticeship training will be recruited to participate from 12 training centers. Recruitment will be limited to two masonry subspecialties (brick, block). The recruitment strategy and sample size was designed to ensure maximum comparability across apprenticeship training centers and intervention groups. Participants will provide signed informed consent to participate, which will be collected in person at their respective training center. Contact with each apprentice in years one or two of training will be made before consenting using the communication strategy appropriate for each training center (either mail, email or phone call). The research team will follow-up with a visit to the training center after contact is made to consent apprentices who agree to participate. Baseline data collection (described below in the “Measures” section) will also be conducted at this visit.

#### Inclusion/exclusion criteria

No subject approached for recruitment into the study will be excluded from participation because of co-morbid medical conditions. We anticipate inclusion of women, minorities, and persons between 18–21 years (i.e., those meeting the National Institutes for Health definition of children) in the study sample. Apprentices who do not have reliable access to a computer or who do not use a cell phone with internet accessibility will be excluded from participation.

### Intervention

To develop the SAVE training, key informants identified by the Masonry r2p Partnership were engaged in focus groups. These key informants included IMI instructors, BAC advisors, ICE representatives, and masonry contractors. We conducted five focus groups with 8–10 key informants completing a needs assessment for ergonomic and safety voice topics, developing an integration plan with existing apprenticeship curricula, obtaining feedback on examples from the field, developing experiential activities, and discussing ergonomic solutions. The focus groups were moderated with a script to elicit productive feedback and recorded by the research team. A qualitative analysis was completed to determine main themes elicited from the groups.

We also completed an e-learning feasibility survey with a randomly selected sample of approximately 35 first and second year apprentices recruited from apprenticeship training centers. This survey was used to determine apprentice use of and access to computers, the internet, smartphones, email, and text messaging and other social media, which will be essential to specific elements of the SAVE training. With input from the focus groups and the feasibility survey, the research team is developing: 1) primary (basic) training delivered by e-learning and face-to-face using a blended learning approach, and 2) secondary (refresher) training delivered by text messages or email. SAVE training will be pilot tested at two training centers, before being tested in the trial.

#### Primary training

Brief, seven to ten minute, e-learning training units will provide apprentices with basic knowledge of the concepts outlined in Table 2. These units will include text, brief video clips, and interactive on-screen slides that guide apprentices through content with brief quizzes that assure understanding [31, 34, 35]. Units are being developed with Captivate (Adobe Systems Incorporated, San Jose, CA) software [13]. Members of the research team have developed similar ergonomics training programs and taught ergonomics with e-learning [36, 37]. Apprentices will be reminded to complete home units by a text message or email in the week prior to onsite training.

**Table 2:** Safety Voice for Ergonomics (SAVE) program content

Ergonomics content	Safety voice content
Introduction to ergonomics	Introduction to safety voice
Anatomy and neutral postures	Rights and responsibilities
Cumulative trauma	What’s the Issue?
Awkward postures	Get advice
Lifting	Choose your goal
Prolonged and repetitive activities	Communicate
Solutions: engineering and administrative	Conflict resolution
Solutions: work practices	

Once at the training center, learning gained from completing the e-learning units will be reinforced by IMI instructors who will provide short (10–15 min), applied, face-to-face shop activities. The primary training will be delivered to apprentices in years one and two of their apprenticeship training in order to allow follow-up for a year before they become journey level workers.

Interactive, face-to-face, problem-solving “shop” and classroom activities will complement the e-learning units and will include vignettes to promote discussion of hypothetical worksite cases related to ergonomics and safety voice. These activities will be integrated with existing apprenticeship training skills. For example, if apprentices are learning about mast climbing scaffolding, the applied shop activity related to ergonomics might relate to keeping the work between the knees and the shoulders. The safety voice activity might be how best to talk with a coworker who does not want to stop work to adjust scaffolding. These vignettes will be brief to fit into existing training time demands.

#### Secondary training

Refresher training for ergonomics and safety voice will be developed to bolster concepts learned by apprentices in the primary training. This training will be delivered through text messages or emails. However, delivery may include Facebook or Twitter depending on participant preference. Secondary training is considered medium engagement learning since apprentices will be asked to respond to text messages or emails. Refresher training will be delivered four times a month over a one-year period to maximize knowledge retention [28, 31].

Secondary training will be an abbreviated version of primary concepts (Table 2), amenable to delivery by text or email messages that are monitored by the researchers. For example, an ergonomic-related text message would state, “During the past week have you worked on adjustable scaffolding?” The apprentice will respond “Yes” or “No.” Apprentices will then receive a follow-up text/email reminding them that keeping work between shoulders and knees reduces wear and tear to their muscles and joints. As a safety voice example, a text message would state, “During the past week, did you speak up to a coworker or supervisor about something you saw that was unsafe?” This would be followed up with, “Speaking up if you see an unsafe work situation can keep you and your coworkers from getting injured.” The texts/emails are meant to reinforce concepts as well as obtain descriptive information about the use of ergonomic interventions in the field.

### Measures

Participants will be asked to complete self-administered questionnaires. At baseline, apprentice participants will complete a demographic questionnaire. At baseline, at the conclusion of primary training, and then at six and 12 months post-primary training (follow-up), participants will also complete health questionnaires (musculoskeletal symptoms and general health perceptions) as well as training content questionnaires. Similar measurement intervals have been used by other investigators to assess durability of training [33, 38]. All training groups (intervention and control) will be measured at the same intervals with the same study instruments. The questionnaires and outcome measures are described below.

#### Demographics

We will obtain information about demographic characteristics (e.g., age, race, gender, education,), personal health (e.g., diabetes, rheumatoid arthritis, prior MSDs), and history of injury or trauma to the upper extremities and spine. The participant's apprenticeship level and other job history information will also be obtained.

#### Musculoskeletal symptoms

The Modified Nordic Questionnaire assesses self-reported musculoskeletal symptoms [39]. It is validated and is among the most frequently used MSD outcome assessment instruments used in ergonomic research [40–42]. The questionnaire requires a “yes” or “no” response to the following three questions for nine different anatomic sites (neck, shoulder, upper back, lower back, elbow, wrist/hand, hip/thigh, knee, feet): “During the last 4 weeks have you had a job-related ache, pain, discomfort”; “During the last 4 weeks have you been prevented from doing your day’s work due to this condition?” and “During the last 4 weeks have you seen a physician for this condition?” The questionnaire takes about three minutes to complete.

#### Health status

The Short Form-12 Health Survey version 2 (SF-12v2®) is a standardized health questionnaire of perceived physical and emotional health status. The SF-12v2 has 12 items asking about the past 4 weeks of physical and emotional health and the impact of their health on physical activities, daily work, pain, energy, and social activities. These are measured on a five-choice response scale from 1 (All of the time) to 5 (None of the time).

#### SAVE knowledge acquisition

These questions will assess knowledge gained of ergonomic and safety voice principles covered in the SAVE training (Table 2) using multiple-choice questions.

#### SAVE safety voice, compliance, participation, and motivation

These questions will evaluate apprentice attitudes and perceptions about safety, using a visual analog scale where participants place a mark on a line anchored with 0 % = Never and 100 % = Always. Safety voice questions include, “Speak to co-workers at risk and encourage them to fix safety problems” or “Tell my foreman about hazardous work.” Such questions have been demonstrated to be valid for evaluating safety voice in youth [43]. Measures of safety compliance and participation have also been used in the construction industry and these will be included as questions [44], using the same visual analog scale, with statements such as, “I appropriately report injuries, accidents and illnesses” and, “I speak up and encourage others to get involved in safety issues.” Finally safety motivation questions will be asked, measured using a scale from 1 (strongly disagree) to 5 (strongly agree) and statements such as, “I feel that is worthwhile to put in the effort to maintain or improve my personal safety” and, “I believe that is important to reduce the risk of accidents and incidents in the workplace” [45].

#### SAVE adoption

Perceived attributes and detractors (barriers) of ergonomic interventions influence whether solutions are adopted by end-users. Based on previous questionnaires developed by the project team [46], we will collect data about level of intervention use, and attributes and detractors to adopting ergonomic interventions. The post-training data collection will provide information about whether apprentices use interventions more frequently, or are more willing to adopt new ergonomic interventions when appropriate. For example, “When working with 12 in. block do you work as part of a lift team?” [47].

#### SAVE reaction

To measure the reaction of the apprentices to the SAVE training, four, standard, 5-point scale questions will be used that are measured only post training (1 = strongly disagree to 5 = strongly agree): 1) I really enjoyed participating in the SAVE training, 2) The SAVE training was extremely useful for improving my health and safety, 3) I changed one or more behaviors as a result of participating in the SAVE training, and 4) I would highly recommend the SAVE training to other apprentices.

### Implementation

#### Train-the-trainer program

A manual describing the SAVE training will be developed for IMI/BAC apprenticeship instructors. This manual will outline logistical issues related to training, such as student and instructor access to the SAVE online materials, detailed instructions for accompanying class/shop activities, curricular integration, and interaction with the investigators. Additionally, the manual will detail ergonomic principles as applied to masonry. Depending upon feedback from IMI instructors, members of the research team will conduct live webinars or in-person Train-the-Trainer classes. Since much of the content of SAVE is experiential training in the shop, it will be necessary for instructors to practice delivering SAVE’s hard and soft skills in the context of masonry skills with feedback from the researchers.

Intervention training, adherence, and fidelity monitoring of the instructors will be the responsibility of the research team. This will be accomplished with regular phone calls and emails to instructors during apprenticeship training classes to assure they are continuing to promote use of SAVE in conjunction with their training programs. This connection will also be used to obtain ongoing feedback from instructors on barriers and facilitators to apprentices integrating knowledge from SAVE into their work habits.

### Statistical methods

Data analyses will assess the *central* hypothesis that the combination of ergonomics and safety voice training will be more effective (as reflected in each dependent measure) than no training or either ergonomics training alone or safety voice training alone.

#### General analytic approach

Preliminary analyses will include inspection of descriptive statistics and features of the data to determine whether data transformations for non-normal data are necessary. We will initially test whether baseline demographic characteristics and dependent variable scores are comparable between the four study groups using univariate analyses of variance or Wilcoxon rank-sum tests for continuous variables, and chi-square tests for categorical variables. Groups will be considered imbalanced on variables that differ at *p* < .10, and all such imbalanced baseline demographic factors will be included in primary analyses as covariates.

We intend to measure the dependent variables at four time points. Because repeated measurements on individual subjects tend to be correlated and, in some cases the number and intervals of time between observations may vary among subjects, and because the design uses clusters of apprentices within training programs, we will analyze each dependent measure with a general linear mixed model (GLMM) for clustered repeated measures data with modeling for 3 independent variables. These variables are 1) Ergonomic Training Group (Yes, No), 2) Safety Voice Training Group (Yes, No), and 3) Time of Assessment (baseline, post-primary training, and six and 12 months post-primary training) with covariates represented in the analyses as necessary, and between subject variability modeled as a random effect. GLMM will account for dependence in repeated measures and accommodate correlated errors, unequal correlations among time points, unbalanced data resulting from missing data points, and unequal intervals between testing occasions.

All analyses will be conducted based on the intention-to-treat principle in which any participant randomized to a treatment group remains in it regardless of adherence to or completion of treatment. We will measure level of participation and conduct a sensitivity analysis that assesses the stability of the conclusions from the GLMM analyses against an available-case analysis that considers only data from fully-adherent participants in a General Linear Model (GLM) repeated measures analysis of variance. The planned analyses involve multiple comparisons, which increases the likelihood that any single outcome will be found to be statistically significant based on chance alone. In order to buffer against potential inflation of type I errors due to multiple tests being performed, we will employ a more stringent type I error criterion (*p* = .01) than the typical .05 criterion.

#### Sample size and statistical power

We have access to 25 IMI apprenticeship training centers from which to recruit. Our plan is to recruit 12 centers to participate in the cluster randomized trial (3 assigned at random to each group) with an average of eight apprentices recruited per center for a sample size per group of 24 (total study sample size = 96 apprentices). Averaging recruitment of eight per center will allow us to extend enrollment beyond eight apprentices at some centers to accommodate for centers that have a total apprentice class size less than eight.

The significance of clustering on sample size estimates is that apprentices within a center have greater resemblance to each other than to apprentices at other centers, resulting in intracluster correlation (ρ). Intracluster dependence will not affect point estimates for study outcomes, but may spuriously narrow confidence intervals within each study group when clusters are appropriately accounted for in statistical analyses [48]. To avoid statistical power problems, we made adjustments to standard power estimates for a 2 x 2 x 4 non-clustered design by employing an inflation factor that accounts for the intracluster correlation in each study group, 1 + (m − 1)ρ where m is the average number of apprentices per cluster [49]. We were unable to find published evidence of intracluster correlation coefficients for outcomes within clusters; therefore, to account for correlation of outcomes within a cluster, we used a conservative estimate for ρ of 0.2 in inflation factor calculations. Given an average cluster size of eight apprentices, the inflation factor is 2.4.

Therefore, to assure that a test of means in the clustered design had sufficient power to establish superiority of any of the intervention groups over the control group, the sample size in standard power calculations was reduced by a factor of 2.4, resulting in a total study sample size of 40 for non-clustered power estimates, and a per-group sample size of 10. Effect sizes for determining power depended on information from a NIOSH systematic review examining the effectiveness of occupational safety and health training [31]. This review established that the across-study standardized mean difference between intervention and control groups post-training for the outcome measure of knowledge was 2.52, for attitudes and beliefs (e.g., self-efficacy, perceived risk, outcome expectations, behavioral intentions) was 0.84, and for behaviors (e.g., hazards and exposures under the worker’s control) was 1.09. Using the lower-bound effect size of 0.84, standard power calculations for a 2 x 2 x 4 design with repeated measures and a conservative estimate of correlation among repeated measures of 0.2, power (1 – β) to achieve statistical significance at a 2-sided type I error rate of .01 was 0.98 for the standard non-clustered design. Even with attrition averaging three apprentices per group (for a total sample size of 28), power would only be reduced to 0.96. In the clustered design, this would equate to a per-group sample size of 17. Given such robust estimates of power for a non-clustered design, we are confident that our clustered design with a sample size per group of 24 (with room for attrition to an n of 17) will have sufficient statistical power to detect significant differences among groups in statistical analyses that appropriately account for the effects of clustering. Power estimates were conducted with G*Power v. 3.1.7 [50].

## Discussion

Masons have the highest rate of overexertion injuries among all construction trades, the highest rate of repetitive bending and twisting, and exceed the construction industry average for nonfatal injuries resulting in time away from work [3]. Ergonomic solutions are the primary method of reducing these exposures and resulting injuries; however, many construction workers lack knowledge about ergonomic principles and solutions, and how to responsibly broach these topics in the hierarchical construction culture. Masonry apprentices are an optimal population for receiving SAVE training as they embark on their careers. The knowledge and skills these workers gain can assist them throughout their careers to minimize the cumulative exposure that leads to MSDs and to lead the construction industry in proactively working more safely. The SAVE project develops and tests an innovative approach to ergonomics and safety voice training that currently does not exist in the masonry trade or the construction industry.

The major strengths of our study are the use of blended learning methods, and the engagement of masonry instructors and the Masonry r2p Partnership in the SAVE project to develop and broadly disseminate a relevant apprenticeship training program. Another strength is the use of a cluster-randomized controlled trial design to accommodate regional training center differences, as well as a repeated measures design to evaluate the change in knowledge, attitudes and beliefs, and behaviors of the apprentices over time. We expect to face challenges with implementing the SAVE program with the diverse training schedules.

We expect to face potential threats to validity due to loss of follow-up and contamination. However, our team has over 15 years of experience conducting ergonomic and intervention effectiveness research in the construction industry and will do our best to minimize these threats. Analyses will be conducted examining differences between those lost to follow-up and those remaining in the study to assist in assessing the extent to which participants may differ from the target population. The threat to validity from contamination (i.e., the unwanted adoption of the interventions by the referent group) is addressed by implementing a single intervention per cluster (training center). Since the interventions require an infrastructure under the control of the investigators, it is unlikely that participants will be able to adopt the intervention without explicit assistance by the research team, preventing meaningful contamination.

From this work, we will develop evidence-based ergonomics training that is integrated with current apprenticeship curricula and disseminated throughout the masonry industry in the United States. The SAVE project will provide innovative training to a new generation of skilled masonry workers who are better equipped with evidence-based strategies for protecting their musculoskeletal health by working more safely.

## Abbreviations

BAC: international Union of Bricklayers and allied craftworkers
FTE: full time equivalents
GLMM: general linear mixed model
GLM: general linear model
ICE: international Council of Employers of Bricklayers and allied craftworkers
IMI: international Masonry Institute
MSD: musculoskeletal disorder
NIOSH: national Institute for occupational Safety and Health
OSHA: occupational Safety and Health Administration
r2p: research to practice partnership
SAVE: Safety Voice for Ergonomics
SF-12v2®: short form-12 health survey version 2
US: United States
